# A qualitative analysis of participants’ reflections on body image during participation in a randomized controlled trial of acceptance and commitment therapy

**DOI:** 10.1186/s40337-016-0120-4

**Published:** 2016-12-12

**Authors:** Maria Fogelkvist, Thomas Parling, Lars Kjellin, Sanna Aila Gustafsson

**Affiliations:** 1 University Health Care Research Center, Faculty of Medicine and Health, Örebro University, S-huset, vån 2, Box 1613, 701 16 Örebro, Sweden; 2 Department of Psychology, Uppsala University, Uppsala, Sweden

**Keywords:** Acceptance and commitment therapy, Body dissatisfaction, Body image, Content analysis, Eating disorders, Qualitative research

## Abstract

**Background:**

Negative body image is a risk factor for development and relapse in eating disorders (ED). Many patients continue to be dissatisfied with their body shape or weight after treatment. This study presents a qualitative analysis of written reflections on body image from patients with an ED and a negative body image before and after an Acceptance and Commitment Therapy group treatment at a specialized ED-unit.

**Method:**

Before and after the treatment participants (n = 47) answered a questionnaire with open ended questions on their thoughts on body image. Data were analyzed through conventional content analysis.

**Results:**

Body image meant different things for different participants. For some it had to do with how you evaluate your body, whereas others focused on whether their body image was realistic or not. Some emphasized their relationship with their body, while some described body image as strongly related to global self-esteem. These different views on the concept of body image affected the participants’ descriptions of their own body image, and how they wanted it to change. Body image was considered a state that fluctuated from day to day. After treatment the participants described changes in their body image, for instance perceiving oneself as less judgmental towards one’s body, and a shift in focus to the important things in life.

**Conclusions:**

The participants had different views on body image and how they wished it to change. Thus treatment interventions targeting negative body image needs to address various aspects of this complex construct.

**Trial registration:**

This study is part of an RCT registered 02/06/2014 in Clinical Trials, registration number: NCT02058121.

**Electronic supplementary material:**

The online version of this article (doi:10.1186/s40337-016-0120-4) contains supplementary material, which is available to authorized users.

## Plain English summary

Many patients with an ED have difficulties with their body image, and struggle to change it. Body image issues are something many people experience, but for a person with an ED it is linked to poorer treatment outcomes and relapses after treatment. In this article we asked patients with an ED to write their reflections on body image. What body image is, how they perceived their own body image, how they wanted it to change, and whether they perceived any changes after a treatment intervention. We found that body image meant different things for different patients. Some described that body image meant how they evaluated their bodies. Some described whether their perceptions of their own bodies were realistic or not. Some described their relationship to their body and others how it was linked to their self-esteem. There were also differences in how they wished their body image to change. Many found that their body image changed after treatment. They still evaluated themselves negatively, but found that they could focus more on other areas in life that felt more important.

## Background

Body dissatisfaction has been defined as “discontent with some aspect of one’s physical appearance” [1], and is a risk factor both for developing an eating disorder (ED) [2] and for relapse after remission from an ED [3]. Although there have been advances in research and the treatment of ED, many patients do not remit despite receiving treatment in accordance with evidence-based practice [4, 5]. Even patients who clinically remit from an ED often continue to be dissatisfied with their body shape and weight [6]. This dissatisfaction can be seen as part of a normative discontent [3], but is nevertheless associated with a risk of relapse to an ED [7] and poor quality of life [8]. Thus, there is a need to further develop or adapt interventions that may increase the remission rate, address persistent body image problems and reduce relapse rates.

Body image is a complex concept, and according to Cash [9] “It encompasses one’s body-related self-perceptions and self-attitudes, including thoughts, beliefs, feelings, and behaviors”. But, for those afflicted with negative body image, what does it mean to have a negative body image, or to be dissatisfied with one’s body? There are numerous studies investigating different ways of measuring aspects of body image [10, 11]. However, we only found two studies that explored patients’ views on body image with open questions. In a study by Espeset, Nordbø, Gulliksen, Skårderud, Geller & Holte [12] they explored the views of body image in different contexts in the daily lives of patients with anorexia nervosa (AN). They found that the participants’ descriptions could be divided into a subjective or objective reality. Depending on how they integrated these two concepts they were categorized as integrated, in denial, dissociated or delusional. These categories were suggested to be used as a continuum to classify severity of body image disturbances, where delusional is the most severe form. They also found that some participants described their body image as stable, while others found it to fluctuate depending on context. The same research team continued to investigate the fluctuations of body images among patients with AN [13]. They concluded that this fluctuation was possible due to an uncertainty about their objective appearance, and found four different contextual cues that made their body image fluctuate; ‘Eating food’, ‘Body awareness’, ‘Emotional experiences’ and ‘Interpersonal influences’. These two studies are, to the best of our knowledge, the only ones that have probed patients’ views on the concept of body image. We were unable to find any studies on patient’s thoughts on how they would like their body image to change due to treatment. In order to target body image in a treatment intervention for patients with an ED it is of importance to find out what a negative body image means to them, what they wish to change in order to feel more content, and whether they perceive that their body image is responsive to treatment interventions. In this study patients with an ED who took part in an acceptance and commitment therapy (ACT) group intervention focusing on body image answered a questionnaire consisting of three open questions about body image at treatment onset and at treatment outset.

Cognitive Behavioral Therapy (CBT) is currently the treatment of choice for bulimia nervosa (BN) and binge eating disorder (BED), and is widely recommended by different evidence-based treatment guidelines (e.g., [14, 15]). An enhanced form of CBT has been adapted to target aspects of all of the ED, with promising results for both adults and adolescents [16–18]. CBT for ED has however been criticized for focusing too much on the content of the patient’s cognitive rather than emotional experience [19]. ACT provides an approach that focuses on accepting unwanted thoughts and feelings, seeing them as part of being human [20]. Preliminary data supports ACT as an intervention targeting EDs and body image issues, where ACT has shown to decrease eating pathology [21–24] and to be feasible and well-tolerated even for patients with chronic and severe AN [25, 26].

Recent CBT programs for ED have implemented targeted body image interventions as part of the overall treatment intervention [27, 28]. Many of these include psychoeducation, awareness and questioning of the thin-ideal, exposure exercises, mindfulness or relaxation techniques, and cognitive restructuring [29–33]. The application of an intervention targeting body image issues with standard CBT might help patients to improve more and even reach levels of ED and body image pathology comparable to those of a healthy population, as suggested from a trial in which a virtual reality intervention was added to standard CBT [34]. Another trial investigated mirror exposure also as complement to treatment, and found reductions in body avoidance behaviors [35]. It has also been suggested that patients might benefit from a shift of focus toward positive aspects of their body image [36, 37]. Though these approaches differ in content and target different EDs of different severity, the literature suggests that an increased focus on body image might help patient reach full recovery from their ED, and perhaps different approaches fit different patients.

Since body dissatisfaction might be viewed as a normative discontent, it is possibly an unsolvable issue. It has been suggested that ED behaviors, such as those involved in over-evaluation of weight and shape, might provide the patient with a means of avoiding disturbing internal experiences such as rejection, imperfection and failure [21]. ACT conceptualizes psychological distress as stemming from the avoidance of unwanted thoughts and feelings, which is then also applicable to ED. Instead, ACT enables and helps to direct attention to what patients want their lives to be about. Since body image problems prompt people to take actions to avoid negative thoughts and feelings by dieting, exercising, body checking and other short-term avoidance strategies, ACT can be hypothesized to be a potent treatment for body image problems.

The aim of the present study is to investigate participants’ thoughts on body image; what body image is (before and after treatment), descriptions of their own body image (before and after treatment), how they would like their body image to change after treatment, and how the participants’ body images have changed after treatment.

## Methods

This study drew its participants from a randomized controlled trial in which an ACT-intervention is compared to treatment as usual for patients with an ED at a specialized ED center in Sweden. The intervention was offered to patients that were already receiving treatment at the ED unit, and who felt that their negative body image hindered their recovery. Participants in the ACT-treatment were given a voluntary questionnaire consisting of three open questions about body image at treatment onset and at treatment outset. (For a fuller description of the intervention see Additional file 1).

### Participants

A total of 53 participants started the treatment intervention, six dropped out before they had participated in half of the sessions, and eight participants attended all sessions. The median number of sessions attended was ten (range 2–12). All the participants were women, in the age range 18–47. Forty-seven participants answered the questionnaire, and were thus included in this study. Thirty-four participants answered the questionnaire before the intervention, and 43 answered after the intervention.

Participants were diagnosed according to DSM-IV [38], but we have chosen to transfer the diagnoses to the DSM-5 [39]. Since the participants had received prior treatment, some of them were relatively free from ED symptoms at group onset and were considered partially remitted, but still had a diagnosis.

Nine of the participants had an AN diagnosis, and nine had a BN diagnosis, five had a BED diagnosis. Other diagnoses were Atypical AN (n = 10), BN of low frequency and/or limited duration (n = 5), Purging disorder (n = 5), and Unspecified Feeding or Eating Disorders (UFED; n = 4).

BMI ranged from 16.6 to 48.5. Four of the participants were underweight (BMI below 18.5), 30 were in a healthy weight span, eight were overweight (BMI 25–30) and five were obese (BMI above 30). Treatment duration (of this treatment episode) ranged from less than a month to 11 years, with a median treatment duration of nine months. For 30 of the participants this was their first treatment episode for an ED, while 17 participants had been in ED treatment previously. At the time of inclusion all participants were treated in outpatient care, but seven of them had previously had inpatient treatment.

### Procedure and measurement

Before and after the intervention, participants were asked to answer a three-item questionnaire. The questionnaire was administered together with several self-report instruments that was part of the RCT trial. However, while the self-report instruments were filled in and returned after the randomization, at a specific meeting, this questionnaire was described as being an voluntary extra survey, and the patients were invited to answer the questions at home, and bring back and leave to the secretary at the unit or to the group leaders at the first group session if they chose to participate. After the intervention patients received the questionnaire at the last group session, and were encouraged to bring it back at the 1 month follow up session, or leave it to the secretary or to the group leaders, if they chose to participate.

Two of the items were identical before and after the intervention, while one was reformulated after the intervention:

- What does the term “body image” mean to you?
- How would you describe your body image today?
- How would you like your body image to change following treatment completion? (Before the intervention)/Has the treatment contributed to any change in your body image? (After the intervention).

### Analysis

Conventional content analysis was used to analyze the answers to these questions [40]. The written reflections were first read several times in order to obtain a general impression. In the next step categories were created based on current data, by marking important statements and grouping data with the same meaning into codes. The answers to each question were analyzed separately. Two researchers (MF and SAG) undertook this step together. One researcher made a preliminary categorization of each question, and created preliminary codes. The other researcher then coded the statements based on the preliminary codes, and the codes were compared in a process of negotiated consensus. Finally each code was discussed and labelled. An answer by one participant may be found in several of the categories, since the written statements are divided into meaning units before categorization. The following is an example of one participant’s response to how she would like her body image to change following treatment completion.

*I would like to be able to look at myself in the mirror and think that what I can see is a normal, fit and healthy person and not need to analyze every aspect of my body to pieces. Instead of battling with my body all the time, I want to love it and value it highly and look after it instead of subjecting it to different diet inclinations and methods like reducing the amount of food and doing more and more physical exercise. I want to feel that I am exercising to be fit and strong and avoid illnesses in the long run rather than exercise being a way of helping to lose weight*. (BN, 23).

In this text, three different codes were identified, divided into three different categories; an appraisal of oneself and one’s body, “*think that what I can see is a normal, fit and healthy person*”, a shift in focus to other areas in life, “*I want to feel that I am exercising to be fit and strong and avoid illnesses in the long run*”, and reducing ED behaviors “*instead of subjecting it to different diet inclinations and methods like reducing the amount of food and doing more and more physical exercise*”.

## Results

The results are presented question by question in the tables below. Since the participants answers on the first two questions were very similar both before and after the intervention, these answers are represented in one table each, including answers from both before and after. Each table presents categories derived through the analysis. Each category is followed by a summary explaining the overarching content of the category, and a quotation that exemplifies the category. Each quotation is followed by information on the ED diagnosis and age of the woman cited, where AN refers both to AN and atypical AN, and BN refers both to BN and BN of low frequency and/or limited duration. In Table 4 there is a column with comments regarding the differences described by the participants.

### What does the term “body image” mean to you?

Participants described the term body image similarly before and after treatment. Many described it as simply as: “*How I perceive my body*”. Some described how they experienced or appraised their body, which we interpreted as synonyms, while others gave a richer description. We identified four categories (see Table 1).

**Table 1:** Participant’s description of the term body image

Category	Summary	Quotation
How you evaluate your body	The body is described for instance as good or bad, attractive or ugly. Participants compare themselves with others. Evaluations are influenced by social ideals, for example being slim is automatically valued as something positive.	*Have had it drummed into me since I was little that slim = happy and successful*. (AN, 41)
If impression is realistic or not	If impression fits in with what others see or with reality.	*What my body really looks like and not the way I think it does.* (Purging disorder, 20)
What relationship you have to your body	Different descriptions of whether the body is shown respect or taken care of. Degree of happiness with body.	*How well you know your body and what is important for it.* (AN, 18)
How your impression fits in with your self-esteem	Impressions govern assessments of self-image and self-confidence	*If I am worth anything or just rubbish.* (BED, 39)

### How would you describe your body image today?

Six concordant categories were identified in the evaluations before and after treatment (Table 2). Participants described a change in their body image after the intervention. This change was described in terms of the categories identified, for instance as perceiving oneself as less judgmental of one’s body or having a more realistic view of one’s body.

**Table 2:** Participant’s descriptions of their own body image

Category	Summary	Quotation
Evaluation of body/body image	Some parts of the body are described with dissatisfaction. Body image is affected by social ideals. Participants compare themselves to ideals and to friends.	*On the whole it is very negative but I have still learned as well to like my body in a different way than I used to.* (AN, 33)
Feelings about your body/body image	Dislike of the body. Feelings of anxiety when thinking about the body. Feeling uncomfortable with the body. Feeling nausea and disgust about the body.	*Don’t really like myself, don’t know what pigeonhole to put myself in – plump, fat or grotesquely elephantine troll-like fat.* (BED, 39) *I feel ok with my body but still not entirely comfortable.* (AN, 18)
If impression is realistic or not	Description of body image as realistic, unrealistic or wrong. Having a realistic image does not mean that participants are pleased with it.	*Sometimes feel that I am fat, which is incorrect. That means that it isn’t true, my weight is normal.* (BN, 26) *Good I can see what I look like even though I’m not satisfied with it.* (Purging disorder, 20)
Changes in relation to body/weight	What participants would like to change about their body, like losing weight or being fitter. Worry about the body changing (gaining weight).	*I really long to feel more muscular, both for my own well-being and also because I think I look better when I am fit.* (UFED, 24) *I’m afraid of putting on weight and being fat.* (AN, 18)
Impressions are inconstant	Body image is affected by feelings and thoughts. The image oscillates between being comfortable with one’s body and appearance to feeling disgust and revulsion and wanting to change.	*Like a roller-coaster. Some days my body feels fantastic, both physically and mentally. But then there are days when I can see everything that’s wrong with it. Then it feels like I’ve taken a step backward, but never back to square one.* (BN, 19)
Thoughts about the body and weight take a lot of room	Appearance is important, which gives rise to thoughts about weight, figure and diet. This focus can lead to discontent, greater concentration on appearance than on well-being.	*I focus more on looking good than feeling good, when it comes to the inside and outside of my body.* (BN, 19) *Don’t think so much about my body today. Not everything is about me any longer.* (Purging disorder, 23)

### How would you like your body image to change following treatment completion?

A recurrent topic in the different categories was the participants desire to be healthy and to accept one’s body the way it is. They expressed a wish to let go of the thoughts and behaviors that are connected to the ED, and to be able to love their bodies. Seven categories were identified (Table 3).

**Table 3:** Participant’s description of desired changes in body image

Category	Summary	Quotation
How you evaluate yourself and your body	A desire to like oneself and one’s body more, see oneself as good enough, to be less judgmental and able to describe the body more neutrally.	*I would like to feel that my body is OK and view it positively instead of something negative.* (Purging disorder, 20) *I would like to be pleased with my body no matter what day it is, how much exercise I have had or what I have eaten during the day. I want to be pleased and happy and feel strong, no matter what.* (AN, 18)
A more realistic body image	A desire to be less judgmental and more accepting of the body.	*That I know what I look like and accept it*. (BED, 44) *I want to be able to see/perceive myself the way I am and to allow/permit myself to feel good.* (AN, 37)
Being influenced less by media or what others think	A desire to create distance from the ideal conveyed through the media and not to have to consider what other people think.	*Not having to make an effort to fit in with an ideal that actually doesn’t exist.* (AN, 37) *And above all I want to be able to look the way I want without having to wonder what others will think.* (AN, 33)
A shift of focus to other things in life	Allowing other areas of life to occupy more space.	*Being able to give priority to things rather than thoughts about my body, allowing myself to put other things first.* (Purging disorder, 23) *For my body not to take up such an important part of my everyday life, I want other things to be more important.* (AN, 24)
Greater respect for your body	Participants express a desire to comply with their bodies’ demands, not have to struggle all the time to oppose changes.	*… and more respect for what my body wants and needs to feel good both inside and out.* (AN, 26) *I want to accept my body as it is without feeling forced to diet/lose weight to feel attractive.* (Purging disorder, 23)
Greater self-confidence or self-esteem	Being able to feel more satisfied and secure in self and appearance. Greater self-confidence or self-esteem.	*I want to feel pleased with myself so that I can be more self-confident.* (AN, 20) *I wish I had faith in myself and more self-esteem and self-confidence*. (BED, 29)
Reducing behaviors linked to eating disorders	Stop avoiding the kinds of things that are really desired. More focus on experiencing activities than on change. Stop behaviors that do not contribute to well-being.	*To be able to live with my body in a better way than avoiding things.* (AN, 25) *Dare to display more, not baggy clothes, being proud of the way I look. Not squeezing my body and not worrying about weight.* (AN, 18)

### Has the treatment contributed to any change in your body image?

There were 38 (88 %) participants that described that the treatment contributed to a positive change in their body image, and many gave a further description of this change. We identified six different categories (Table 4). The quotation below is one example of how lavish a description could be.

**Table 4:** Participant’s descriptions of perceived changes in their body image after the intervention

Category	Summary	Quotation	Comments
Different evaluation of body	The body is described more objectively, less critically. Evaluations do not have the same kind of impact. Greater awareness of how the body is objectified by self-scrutiny.	*I’ve learned that you don’t have to evaluate all the time, see it as a description.* (AN, 18) *I don’t evaluate my body only as bad and ugly but still feel that I am too fat.* (AN, 26)	Being less judgmental does not mean that the participants are automatically happy about the appearance of their body.
New insights have been acquired or new ways of thinking	Greater understanding of the body, ways of thinking and the ED. Awareness of thinking errors. Insight into devaluation of self. Insight into how well-being declines when feelings focus on the body and how this can be influenced.	*I have learned so much about the basis for why I think as I do and then it is easier to know what to do to like my body.* (AN, 18) *I have understood that irrespective of whether we are slim or fat we have similar thoughts and feelings. And that I’m the one that devalues myself, I am my own ‘hooligans’.* (BED, 29)	Participants describe how they have become more aware of different factors that both sustain and resolve their views, which in its turn creates possibilities of more flexible action.
Acquired help to change behaviors	Tested things previously avoided through fear. Lifestyle changes.	*Discovering alternative behaviors for all the evasions I had previously focused on.* (BN, 28) *Plus I have become better at doing things even though they go against the grain and I don’t feel comfortable with my body.* (BN, 22)	Participants describe a wider repertoire of behaviors even though they feel uncomfortable.
Greater distance to thoughts and feelings	Beneficial to see thoughts as thoughts or feelings as feelings. Importance of being in the here and now and not being bogged down in old patterns of repetitive thinking.	*That I don’t allow my feelings to magnify things so much, a feeling is “only” a feeling and a thought is “only” a thought. Nothing is true until I allow it to be.* (AN, 40) *I have really learned that I don’t have to like my thoughts but they can still be there anyhow.* (BN, 28)	Participants describe how they still have the same thoughts as before but have learned a new relationship to them when they crop up. This also creates possibilities of more flexible actions.
The work on values is described as having helped to shift focus to the important things in life	Values are embraced and influence choice of behaviors.	*… realize instead that it’s things like work/studies, friendship, relationships etc. that make all the difference to how I feel and what kind of life I lead.* (UFED, 24)	Participants describe how working with values has helped them to focus on other things in their lives than ED.
A change in the relationship to their bodies	Greater appreciation of the body. Good enough as they are. No longer as self-destructive. More attention paid to bodily needs.	*I have to a great extent stopped being “nasty” and making “awful comments to myself.* (BN, 26) *I listen more to what my body needs and show it what appreciation I can.* (BN, 19)	Participants describe a change in both the way they think about and behave toward their bodies.

*I’ve got a more relaxed relationship to my body and my appearance. It doesn’t matter as much any longer what I feel about my body. As I said I’m not happy with it, but it doesn’t take up as much room as it were. Before when I thought about the future there was always a perfect body somewhere in the frame, it really was a strong and fixed idea that my body had to change if I was going to be successful. Now I think differently and realize instead that it’s things like work/studies, friendship, relationships etc. that make all the difference to how I feel and what kind of life I lead. I don’t judge my body as strictly as I used to either, at times I can even feel that I’m OK. It has also become easier not to start judging other people’s bodies and comparing them with mine, like at parties. I think more about the person I am meeting, not whether she is slimmer than me and that that makes me feel even fatter. In some kind of way I can still see that I am pretty “normal”, before I could feel that I was almost some kind of ugly fat troll among my “attractive” friends and acquaintances. Now I can see that there’s nothing odd about my body really.* (UFED, 24)

### Additional comments

In addition to the processes that were specific to this treatment, participants also described more general aspects they had found helpful. Some responded that they enjoyed the group format of the treatment and being able to meet other people with similar thoughts and ideas. *“I’ve realized that I’m not the only one who walks around thinking that I’m not good enough the way I am”* (BN, 46). Some described an increase in their self-esteem, and one participant mentioned a heightened awareness of risk factors.

Five of the participants did not find the treatment helpful or thought it had only been somewhat helpful. One participant mentioned that she had gained new understanding of her difficulties, but she did not notice any changes in behavior. She expressed a need for more time to make changes.

## Discussion

The participants described that they perceived their body image in similar ways before and after the intervention. A majority described changes in their body image after the intervention, or that they continued to be dissatisfied but that it was less important after treatment where they described how other areas in life got more attention. This suggests that body image is perceived as a trait that is responsive to treatment.

The answers of the participants reflected the definition of body image as a complex concept incorporating several components [9]. Some described body image simply as how they evaluate their body. From this point of view, therefore, having a negative body image means that you evaluate your body negatively (i.e., body dissatisfaction). Others answered that body image was linked to whether the body image was realistic or not. A negative body image would then imply a disturbed perception of one’s body. One example can be found in the participant who said that her body image was “incorrect” since she sometimes perceived herself as large although she knew she was of normal weight. Some participants emphasized their relationship with their body, which indicates that a negative body image is related to treating your body without respect, and not listening to it. And finally some participants answered that body image was linked to how their perception affected their self-esteem. Thus, a negative body image would mean that self-evaluation is very dependent on shape and/or weight (i.e., over-evaluation of shape and weight). All these descriptions have been linked to body image previously and they are common features among those with ED [2, 39, 41, 42]. The participants often described several components in their evaluations. For instance the participant who responded that she had a good body image because she could see what she looked like (realistic perception), even though she did not like it (evaluation). Different components might be combined into concepts such as in body image disturbance, which is commonly described as incorporating one evaluative and one perceptual component [41]. It is important to acknowledge that there are individual differences in the conceptualization of body image. These differences might have implications both in planning treatment and assessing body image. For instance, one patient might view improvement in body image as becoming less judgmental while another views it as acquiring a healthy and respectful relationship with her body. These differences impact on what the individual patient wants help with as well as how he or she evaluates the outcome of treatment.

The participants’ descriptions of their body image were on the whole very negative. This is, of course, not unexpected as the treatment focused on patients with remaining body image problems. For example, many participants described how their body image differed in different contexts and was affected by their mood. Several participants described how they sometimes had a realistic body image (for example of being “normal weight”) and at the next moment, due to a change in context, felt very fat. It has been suggested that the body image of patients with AN fluctuates throughout the day depending on the context and that this is a consequence of a failure to integrate the subjective body image with the objective one, the realistic or not aspect [13]. This inability makes patients with AN more susceptible to contextual triggers that make their body image fluctuate. The results of this study indicate that this can be equally true for patients with other ED diagnoses.

From an ACT perspective, the fluctuations in our body image or our self-evaluation due to different contexts could be considered a normal variation. The fluctuation is not something that needs to be addressed or changed and we do not have to remedy these unwanted experiences. Instead the participants are encouraged to be mindful, to accept and to keep moving in the valued direction, directing their attention toward what they want their life to be about rather than what they want to avoid. This is in accordance with a study that suggests that mindfulness might enhance self-acceptance of one’s body and appearance [43]. Another study found a correlation between improvements in awareness, acceptance and emotional avoidance and greater improvement in ED symptoms [44]. Yet another study suggested that acceptance of discomfort enhanced motivation to change ED behaviors [45].

In the answers to the last question before treatment it was clear that the participants wanted to evaluate their bodies less negatively and more realistically, be less preoccupied with their bodies, be more respectful to their bodies and not engaging in ED behaviors, having a more positive global self-evaluation and being less influenced by mass media. From an ACT theoretical perspective it might be hypothesized that present moment awareness could reduce preoccupation/fusion with body image thoughts, and that the work on values-driven action could reduce overvaluation of weight and shape. Mindfulness practice might be hypothesized to help patients to neutrally describe their bodies, and thus reduce perceptual disturbance and the negative evaluations of their bodies.

After the treatment the participants responded that they had been able to try behaviors previously avoided and take steps toward their values instead of avoiding discomfort. They reported that they had adopted a stance of self as context, were able to be more in contact with the present moment and saw thoughts as thoughts rather than truths. They could be more acceptant and descriptive than judgmental toward themselves and their bodies. Many of them still described being dissatisfied with their bodies but emphasized that this was no longer the focus of their attention. This is an important shift of focus, since body dissatisfaction is a normative discontent [46], and for many people probably an unsolvable issue. We interpret this to mean that the ACT-specific skills the participants learned in this study were appropriate and helpful for the participants. The objective in ACT is not to change the suffering that life entails but to be able to act in accordance with values in a flexible way [47]. The current study did not intend to evaluate or asses the effectiveness of the intervention, which would require a different study design. It can only suggest hypothesizes that further studies might give answers to.

In this study we used a questionnaire with open ended questions instead of interviews or collecting data from self-report questionnaires. One reason for this was to capture the informant’s spontaneous reflections yet still collect a comparably large number of evaluations. Another strength of this study is the variation of ages, diagnoses treatment duration and BMI, which increases the transferability of the results to other ED-populations. A limitation in this regard is of course that there were no male participants.

In order to develop effective interventions, we believe it is important to investigate the participants’ reflections, rather than merely collect data from pre-formulated questionnaires. Participants were not asked to evaluate the ACT processes specifically, but they have participated in, and read a book on, a treatment that explicitly targets these processes. This probably influenced their answers. A limitation with written evaluations is that they provide no opportunity to ask questions to clarify statements, which makes it difficult to be sure that it really was specific processes that the participants were referring to. Further, the current method of analysis comprises an element of subjective interpretation when creating and categorizing codes. The texts are, however, quite short and the categories and quotations follow the participants’ wordings closely, so it should be easy for an uninformed reader to evaluate them.

## Conclusions

There were individual differences in the way participants described both the term body image and their own body image. They also described how they wanted their body image to change in different ways. According to our interpretation of the results, participants described that their body image was responsive to treatment. They described that they could relate to their bodies differently, and lead their lives in more accordance with their values. Treatment interventions targeting negative body image needs to address various aspects of this complex construct.

## Additional file

Additional file 1: Description of the intervention. (DOCX 23 kb)
