# Supplementary material for: A qualitative analysis of participants’ reflections on body image during participation in a randomized controlled trial of acceptance and commitment therapy
Source: J Eat Disord. 2016 Dec 12;4:29. doi: 10.1186/s40337-016-0120-4 (PMC5151132; doi:10.1186/s40337-016-0120-4)
Supplement: Additional file 1: — Description of the intervention. (DOCX 23 kb) [file 40337_2016_120_MOESM1_ESM.docx]

**Description of the intervention**

The intervention is based on a book that was written as a self-help manual for people with body image issues [1]. A treatment manual for a group format was created, following the steps in the book. The intervention comprises an individual preparation session followed by 12 group sessions and a 1-month follow-up session (Table 1). Each participant receives a copy of the book. Every session starts with a mindfulness exercise that can be downloaded from a homepage. Between the weekly sessions the participants work with the latest step in the treatment, as well as with mindfulness and values (see contents of the intervention and a fuller description below). Each step is contingent on the next, building on the core processes in ACT. The group size differs between 5 and 9 participants, and there are two group leaders.

**Table 1 The content of the sessions in each step**

| Step | Content | Session |
| --- | --- | --- |
|  | *Individual introduction and preparation session* |  |
| 1 | Values – what is important in your life? | 1-2 |
| 2 | Rediscover your body – beyond your inner dialogue | 3-4 |
| 3 | The relation between body image and self-esteem – what do you want it to be | 5-6 |
| 4 | Delineate your inner dialogue – understand, take a stand, change and accept | 7-8 |
| 5 | Willingness and acceptance – the art of living | 9-10 |
| 6 | Take care of your body and be mindful | 11-12 |
| 7 | Sustain changes and prevent relapse – keep it up | 12 |
|  | *Individual follow up session* |  |

Before the individual introduction session participants read the introduction and the first chapter of the book. The session aims to motivate and prepare the participants for treatment. Participants also have homework to do before the initial group session, further reading and exploratory exercises.

The first step introduces values. The participants formulate values for different areas in their lives, such as work or relationships. Declaring values functions as a compass that points in the direction you want to go in your life, rather than providing explicit goals. An example of a value might be “to create warm, caring, supportive and loyal relationships with my family members”. When participants have formulated their values, they are encouraged to reflect on the extent to which these are their own values, or if they are internalized expectations from others or society. They are then encouraged to reflect over what hinders them from moving in direction of their values: What behaviors have they used that in the short run help them avoid discomfort (e.g. anxiety) but may in the long run lead away from their values? In each step throughout the treatment the participants choose an area of their life to focus on during the following step, and they formulate activities to engage in to move closer to their values in this area. In ACT this is referred to as committed action.

The second step introduces the ACT processes called present moment awareness, self as context and defusion. The principles of avoidance and negative reinforcement are described. The participants learn to recognize situations in their daily life where they are using avoidance and control strategies. The participants record these behaviors in different situations. The book explains how our minds constantly produce thoughts that we are easily caught up in, and describes our minds as a “thought generator”. Every time the “thought generator” produces judgmental thoughts, participants are prompted to gently return to the present moment.

In the third and fourth step the participants explore what they want their self-evaluation to be based on. They formulate a hierarchy of behaviors they usually avoid, and record thoughts and consequences while performing them. This hierarchy of behaviors is used throughout the treatment and is congruent with their previous work on values. Perfectionistic behaviors are addressed, and investigated in terms of whether they take them toward or further away from their values. Participants also examine to what extent their attention is under the control of their inner dialogue, and how judgmental thoughts produced by the “thought generator” affect them in terms of behavior. The participants practice ways of gaining awareness of these judgmental thoughts. They register the thoughts and the behaviors that usually follow them.

The fifth step introduces willingness and acceptance more thoroughly. Willingness is an active choice that involves admitting all thoughts, feelings and discomfort without controlling or belittling them. Acceptance is introduced as the act of taking steps toward values in the presence of willingness. By continuing toward their values in the presence of discomfort, participants’ behavior options increase (i.e. psychological flexibility). This step also describes a difference in the way discomfort can be experienced. There is a universal kind of discomfort felt by all of us when faced with hardships in our lives. We cannot remove or reduce this kind of discomfort. However, if we devote a great deal of effort to trying to do so, it actually often causes additional discomfort. This is exemplified to the participants by an exercise in which they carry a backpack. The further it is held from the body, the heavier and more of an impediment it gets. The backpack symbolizes life’s hardships and accepting the discomfort and embracing it enables us to get in contact with more behavioral options.

Respecting and listening to each other characterize every healthy relationship and the sixth step emphasizes that this also applies to the relationship with the body. The participants record to what extent they show their body respect and care by giving it exercise, sleep, rest and nutrition. This step emphasizes the process of present moment awareness with the body and the investigation of enjoyment.

The seventh step is about sustaining changes and preventing relapses. A metaphor describes the path to values as being the driver of your own bus. You have decided on the direction towards your values, but as you drive toward these values, the passengers on the bus start voicing their discontent. They try to persuade you that you are not good/beautiful/smart enough to make the journey. If you listen to the passengers and go back to your old routes they cool down and stop bothering you. However, this does not take you toward your values. So you decide to make a new try toward a valued life, taking note as the passengers voice their discontent but staying focused on the route you have taken, driving the bus kindly but firmly toward your goal.

One month after the last group session the participants meet with one or both of the group leaders for an individual follow-up.

**References**

1. Ghaderi A, Parling T. Lev med din kropp: om acceptans och självkänsla. Stockholm: Natur & kultur; 2009.
